# Electrochemical and galvanic fabrication of a magnetoelectric composite sensor based on InP

**DOI:** 10.1186/1556-276X-7-379

**Published:** 2012-07-09

**Authors:** Mark-Daniel Gerngross, Jürgen Carstensen, Helmut Föll

**Affiliations:** 1 Institute for Materials Science, Christian-Albrechts-University of Kiel, Kaiserstrasse 2, Germany

**Keywords:** porous semiconductors, InP, piezoelectric

## Abstract

A process chain for a magnetoelectric device based on porous InP will be presented using only chemical, electrochemical, photoelectrochemical, photochemical treatments and the galvanic deposition of metals in high-aspect-ratio structures. All relevant process steps starting with the formation of a self-ordered array of current-line oriented pores followed by the membrane fabrication and a post-etching step, as well as the galvanic metal filling of membrane structures are presented and discussed. The resistivity of a porous InP structure could be drastically increased and, thus, the piezoelectric performance of the porous InP structure. The developed galvanic Ni filling process is capable to homogeneously fill high aspect-ratio membranes.

## Background

The aim of this work is to develop small and cheap magnetoelectric sensors that are capable to sense biomagnetic signals in the range of pico tesla with a high sensitivity. In principle, this can be achieved with multiferroic materials, such as Cr_2_O_3_[1], that show magnetoelectric behavior. The drawbacks of these materials are their small effect magnitude and a Curie temperature far below room temperature[1]. Magnetoelectric composites overcome these problems and are very promising candidates for biomagnetic sensing applications. Magnetoelectric composites consist of a piezoelectric and magnetostrictive component in various geometrical arrangements.

In this paper, a production chain for such a device will be presented using only chemical, electrochemical, photoelectrochemical, and photochemical etching of InP and galvanic deposition of metals. A 1-3 composite geometry is chosen because it allows for very high contact areas between the piezoelectric InP matrix (3-D) and the magnetostrictive wires (1-D). This is another prerequisite for a good magnetoelectric sensor performance. InP as a III-V compound semiconductor belongs to the cubic 4macro3m crystal structure and, thus, in principle allows for strong piezoelectric behavior. Due to its cubic crystal structure, the only non-vanishing piezoelectric coefficient is the _*d*14_coefficient. Unfortunately, no intrinsic, i.e. insulating, InP can be produced, and thus, any piezoelectric voltage is short circuited by free charges inherent in the material. This problem has been overcome by etching an almost hexagonally close-packed array of current-line oriented pores (curro-pores) in <100> oriented InP wafers, leaving a porous structure with completely overlapping space charge regions (SCR), at least after further chemical etching. This allowed for increasing the piezoelectric response by a factor of 30 in comparison to bulk InP [2,3]. Galvanic filling of the pores with a magnetostrictive metal – such as Ni – allows for filling of high aspect-ratio geometries. This enables the use of a several hundred microns thick piezoelectric layer, resulting in a higher magnetoelectric voltage coefficient. Galvanic filling of a membrane structure is much simpler than galvanic pore filling and a well established technique [4-6]. From basic principles, it is clear that it is not possible to etch curro-pores through the complete InP wafer. Thus, the bulk back-side of the InP pore array is opened photoelectrochemically/photochemically after the curro-pore formation. The individual steps of the sensor device production will be discussed as well as some first results of the piezoelectric and electric properties.

## Methods

In this work, single-crystalline (100) InP wafers, doped with S at a doping concentration of 1.1 × 10^17^ cm^-3^ and a resistivity of 0.019 *Ω*cm are used. The InP wafers are double-side polished and epi-ready. Two different wafer thicknesses were used, 400 and 500 µm. The curro-pores were etched in an electrochemical double cell with a 6 wt.% aqueous HCl electrolyte at 20 °C [7,8]. To achieve a homogenous pore nucleation, a voltage pulse was applied followed by a constant anodic potential.

In the second step, the membrane was produced by galvanostatic etching of crystallographically oriented pores (crysto-pores) in the wafer back-side until the previously etched curro-pore array is reached. During formation, this mesoporous layer was simultaneously dissolved photochemically by high power blue illumination. To avoid underetching and to have a good selectivity between the curro-pore array and the bulk back-side, blue light assisted photoelectrochemical etching is used. The etching was carried out in the same electrochemical double cell so that a misalignment due to a cell change is avoided. The etching is done in the same HCl-containing electrolyte at 20 °C. The high power blue illumination is provided by an Enfis UNO Tag LED array (ENFIS LIMITED, Swansea, UK) with a mean wavelength of 470 nm. To control the whole membrane etching process, the voltage was monitored and the Fast Fourier Transform impedance spectroscopy data were recorded, which will not be discussed in this paper.

The third process step is the post-etching of the InP membrane. This step is necessary because the width of the SCR is much larger during the anodic pore formation than at open-circuit conditions [9]. Thus, only anodic pore etching does not result in a porous InP layer with completely overlapping SCR. Therefore, the InP membrane is post-etched in an HF : HNO_3_ : EtOH : HAc containing electrolyte under a cathodic potential for 48 h at 20 °C. The electrolyte was optimized to show isotropic etching behavior – especially along the full length of the high aspect-ratio pores – and to be self-limiting at the SCR surrounding each pore. A cathodic potential was chosen to artificially shrink the SCR around each pore and to expose more area unguarded by the SCR to the electrolyte for dissolution. As the post-etching electrolyte is self-limiting at the SCR, a strongly improved overlapping of the SCR is obtained under open-circuit conditions. The electrolyte is pumped through the complete membrane to ensure homogenous etching over the whole membrane thickness.

In the fourth step, the post-etched membrane shall be galvanically filled with a magnetostrictive material. To prevent possible leakage currents between piezoelectric matrix and magnetostrictive filler, a thin dielectric interlayer consisting of Al_2_O_3_ has to be deposited by atomic layer deposition (ALD). Therefore, the galvanic filling process is developed in an anodically oxidized aluminum (AAO) membrane that is commercially available at Whatman GmbH (Dassel, Germany), with a nominal pore size of 200 nm, which is similar to the average pore size obtained in the here presented InP membranes. A plating base consisting of 25 nm of Ti and 450 nm of Au was sputtered on the back-side of the membrane. The AAO membranes are galvanically filled in a single cell. The working electrode is a Pt sheet, on which the back-side of the membrane is mounted with InGa providing a good electrical contact. A Watt’s type based Ni electrolyte adjusted to a pH value of 2 by H_2_SO_4_ has been used. The electrolyte temperature was held constant at 35 °C. The galvanic deposition was carried out under galvanostatic conditions at a current density of 17 mA/cm^2^.

The etched and galvanically filled nanostructures were investigated using a HELIOS D477 SEM (FEI Co., Hillsboro, OR, USA). The piezoelectric properties of the InP nanostructures were characterized by a double beam laser interferometer from aixACCT Systems GmbH (Aachen, Germany) and the electric properties by a four-point measurement with an Elypor-01 from ET&TE GmbH (Kiel, Germany).

## Results and Discussion

**Figure 1:**
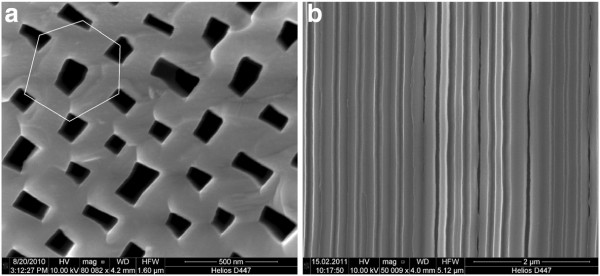
SEM images of electrochemically etched curro-pores in InP. (**a**) Top view and (**b**) cross-sectional view.

The anodic pore formation is optimized to be self-organized with an almost perfectly hexagonally close packed structure as shown in Figure 1a. The pore walls have a mean thickness of around 190 nm. The typical aspect-ratio of the pores is between 3,000 and 3,700 after electrochemical pore formation. The pore shape is trapezoidal, as depicted in Figure 1a. As illustrated in Figure 1b, the curro-pores grow straight in <100> direction.

**Figure 2:**
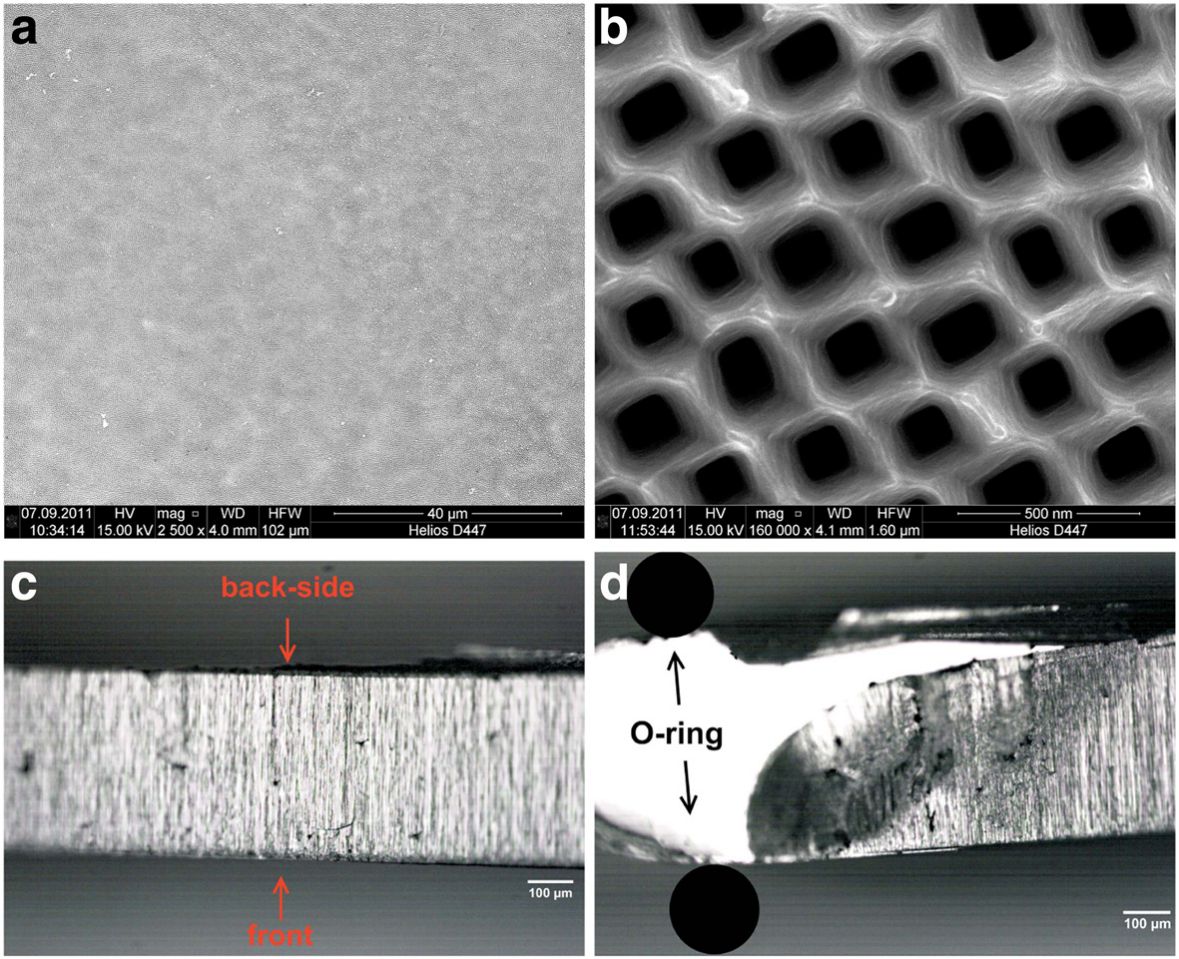
SEM images of the membrane back-side. (**a**) Top-view and (**b**) highly magnified top-view. Optical microscopy images of the membrane in cross-section (**c**) in the center of the membrane, and (**d**) near the O-ring.

Figure 2a–d shows that the previously etched curro-pore array can be opened by photoelectrochemical/photochemical etching. This membrane fabrication process has several advantages. Firstly, the resulting membrane has a great surface homogeneity, as illustrated in Figure 2a and b. The second advantage is that no underetching near the O-ring occurs, as depicted in Figure 2d. The underetching is prevented by choosing photochemical dissolution of the mesoporous layer with blue light. Blue light is used, because it is absorbed very surface near and, thus, surface near dissolution of the mesoporous layer is highly enhanced compared to isotropic dissolution. The third big advantage is that the membrane thickness is freely adjustable from nearly wafer thickness (up to almost 500 µm) to less than 100 µm. This allows the aspect-ratio of the pore structures to be smaller than 1,000, which is especially important for a homogenous coating of pore walls with an Al_2_O_3_ interlayer by ALD as discussed later. The free adjustability of the membrane thickness is a direct consequence of the great surface homogeneity and the absence of underetching phenomena. Fourthly, the membrane fabrication process is semi self-limiting as the dissolution rate drastically decreases as soon as the curro-pore array is reached. The pore openings at the membrane back-side can be modified from cone-like to straight by adjusting the photocurrent due to the applied illumination. A small photocurrent, e.g., results into cone-like pore openings, as depicted in Figure 2b.

**Figure 3:**
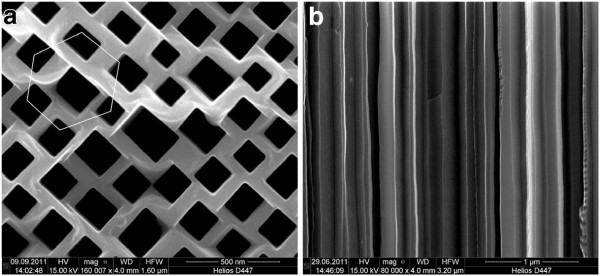
SEM images of the membrane structure after 48 h of post-etching under cathodic bias. (**a**) Top view and (**b**) cross-sectional view in the middle of the membrane.

The pore structure after 48 h of post-etching is shown in Figure 3a and b. Compared to the only electrochemically etched pores, the cathodically post-etched pores are now rectangular with 90° angles and completely straight and nearly equi-distant pore walls. The mean pore wall width decreased from around 190 nm for the only electrochemically etched samples down to around 80 nm after 48 h of cathodic post-etching. The post-etching is homogenous over the complete pore length. This can be seen when comparing the cross-sectional view of the pore structure before post-etching (Figure 1b) and after post-etching (Figure 3b). The electrical characterization of the cathodically post-etched sample exhibits a completely ohmic behavior and an increase in the resistivity to 57 *Ω* cm. Compared to the bulk InP resistivity of 0.019 *Ω*cm, this is an increase by a factor of 3,000. The piezoelectric properties of the porous InP sample have been investigated using a double beam laser interferometer. A linear dependence between the applied voltage and the measured displacement is found - as expected for piezoelectric, but not for ferroelectric materials. The _*d*14_ component is measured to be around ∣60∣ pm/V, which is in the same order of magnitude as sputtered PZT thin films [10] and by a factor of 30 larger compared to bulk InP [2,3]. By luck, the maximum magnitude of the piezoelectric effect is in the <100> direction, which is exactly the optimal growth direction of the curro-pores in (100) oriented InP wafers.

For the magnetoelectric composite device, the galvanic filling with a magnetostrictive metal, such as Ni, is needed. The post-etched InP membranes can be (and already have been) coated with a thin Al_2_O_3_ interlayer by ALD to prevent ohmic contacts between the piezoelectric and magnetostrictive component. Therefore, the galvanic deposition process of Ni can be developed and optimized in AAO membranes with similar pore dimensions because they are cheap and commercially easily available. Afterwards, this Ni deposition process is most probably directly applicable to Al_2_O_3_ coated InP membranes, because the interface electrolyte/Al_2_O_3_ is identical, and the pore dimensions are similar. The ALD deposition process of Al_2_O_3_ is capable to coat membrane structures with an aspect-ratio larger than 1,500.

**Figure 4:**
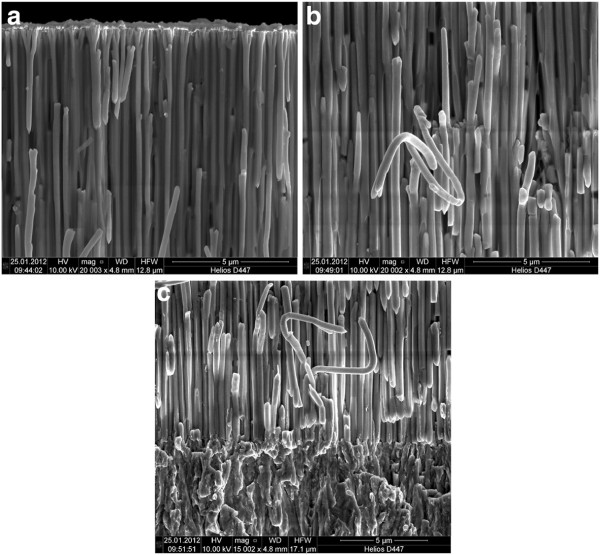
SEM images of AAO membranes with Ni. (**a**) Bottom part of the membrane including the plating base, (**b**) middle part, and (**c**) top of the membrane including overfilled Ni layer.

Figure 4a shows the bottom part of a AAO membrane completely filled with Ni. One can see that the Ti/Au plating base adheres very well to the membrane. The Ni nanowires directly start growing from the plating base. The nanowires are solid and do not show any voids on optical inspection. Some of the Ni nanowires are broken or missing due to the sample cleavage. In Figure 4b, the middle part of the filled membrane is shown. The nanowires are still solid without any voids. They tend to be easily mechanically deformable, as indicated in Figure 4b by the twisty Ni nanowire in the middle of the picture. In order to show the possibility of filling the membrane completely with Ni, the membrane is overfilled resulting in an approximately 7 µm thick closed Ni film on top of the membrane surface. This layer is not caused by single Ni nanowires reaching the top surface faster than others, starting the formation of a solid Ni layer at the surface. It rather seems that all nanowires reach the surface at the same time because there are no empty pores visible, as shown in Figure 4c. The Ni nanowires remain solid even at the surface and do not exhibit any voids, as already seen in the previous parts of the membrane.

## Conclusion

A self-ordered membrane structure in InP with strong piezoelectric behavior has been fabricated using only chemical, electrochemical, photoelectrochemical, and photochemical treatments. By cathodic post-etching of this pore structure, the resistivity could be drastically increased by a factor of 3,000 allowing for a strong piezoelectric effect being 30 times larger than for bulk InP. A photoelectrochemical/photochemical process for fabricating InP membranes with a freely adjustable thickness and high surface homogeneity is presented. The galvanic Ni filling process, which has been presented in this work, will be transferred to Al_2_O_3_ coated InP membranes, and after galvanic Ni filling, the device is expected to show a good magnetoelectric performance.

## Competing interests

The authors declare that they have no competing interests.

## Authors’ contributions

All authors have contributed equally to this work, read, and approved the final manuscript.

## Authors’ information

HF received his PhD degree in Physics in 1976 from the University of Stuttgart in conjunction with the Max-Planck-Institute for Metal Research in Stuttgart. After post-doctoral work at the Department of Materials Science and Engineering at Cornell University (1977 to 1978) and a position as guest scientist at the T.J. Watson Res. Center of IBM in Yorktown Heights (1979 to 1980), he joined Siemens in 1980, working in the newly founded Solar Energy Department of Central Research in Munich. In 1991, after holding various senior positions in microelectronics development at Siemens, he accepted an offer of the Christian-Albrechts-University of Kiel to become the founding dean of the newly established Faculty of Engineering, where he also holds the chair for Materials Science. Since 1998, he is back to research as a tenured full professor, with particular interest in solar cell technology and the electrochemistry of semiconductors. He is one of the pioneers in the field of porous semiconductors, established the so-called ‘CELLO’ technique for characterizing solar cells, and has coauthored more than 250 papers and about 30 patents. He is also well known for his ‘Hyperscripts’ in the Internet (about 1.5 Mio requests per month).

JC began his studies in physics at the Christian-Albrechts-University of Kiel 1984 and received his PhD from the Institute of Theoretical Physics in 1993 for work on superconductivity. Subsequently, he joined the group of Prof. HF at the Institute for Materials Science at the Christian-Albrechts-University of Kiel as a research associate. He began his work employing semiconductor electrochemistry for the mapping of minority carrier life-time in solar cell substrates and in the emerging field of porous semiconductors and related self-organization phenomena. He was instrumental in developing the unique CELLO and ‘CELLOplus’ measurement techniques capable of mapping nearly all of the important solar cell parameters with good local resolution. He has coauthored more than 120 papers.

MDG received his MSc degree in Materials Science in 2010 from the Christian-Albrechts-University of Kiel. Subsequently, he joined the the group of Prof. HF at the Institute for Materials Science at the Christian-Albrechts-University of Kiel as a PhD student in 2010 working on the development of magnetoelectric composites based on porous semiconductors.
